# Supplementary material for: Chemical Characterization of Flowers and Leaf Extracts Obtained from Turnera subulata and Their Immunomodulatory Effect on LPS-Activated RAW 264.7 Macrophages
Source: Molecules. 2022 Feb 6;27(3):1084. doi: 10.3390/molecules27031084 (PMC8839466; doi:10.3390/molecules27031084)

Chemical Characterization of Flowers and Leaf Extracts Obtained from *Turnera subulata* and Their Immunomodulatory Effect on LPS-Activated RAW 264.7 Macrophages

Jefferson Romáryo Duarte da Luz [1,2], Eder A. Barbosa [3], Thayse Evellyn Silva do Nascimento [2,4], Adriana Augusto de Rezende [1,4], Marcela Abbott Galvão Ururahy [4], Adriana da Silva Brito [5], Gabriel Araujo-Silva [6], Jorge A. López [2] and Maria das Graças Almeida [1,2,4,*]

[1] Post-Graduation Program in Health Sciences, Health Sciences Center, Federal University of Rio Grande do Norte, R. Gen. Gustavo Cordeiro de Farias, s/n—Petrópolis, Natal/RN 59012-570, Brazil; jefferson_romaryo@hotmail.com (J.R.D.L.); adrirezende@yahoo.com (A.A.R.)
[2] Multidisciplinary Research Laboratory, DACT, Health Sciences Center, Federal University of Rio Grande do Norte, R. Gen. Gustavo Cordeiro de Farias, s/n—Petrópolis, Natal/RN 59012-570, Brazil; t_hayse_13@hotmail.com (T.E.S.N.); jorgejal@gmail.com (J.A.L.)
[3] Laboratory of Synthesis and Analysis of Biomolecules (LSAB), Institute of Chemistry, Darcy Ribeiro University Campus, University of Brasilia, Brasília/DF 70910-900, Brazil; bioederr@gmail.com
[4] Post-Graduation Program in Pharmaceutical Sciences, Health Sciences Center, Federal University of Rio Grande do Norte, R. Gen. Gustavo Cordeiro de Farias, s/n—Petrópolis, Natal/RN 59012-570, Brazil; marcelaururahy@yahoo.com.br
[5] Faculty of Health Sciences of Trairi (FACISA/UFRN), R. Passos de Miranda, Santa Cruz/RN 59200-000, Brazil; britoas.ufrn@gmail.com
[6] Organic Chemistry and Biochemistry Laboratory, Amapá State University (UEAP), Av. Presidente Vargas, s/n, Centro, Macapá/AP 68900-070, Brazil; gabriel_ar4@yahoo.com.br
* Correspondence: mgalmeida84@gmail.com; Tel.: +55-84-3342-9807; Fax: +55-84-3342-9833

Supplementary material

Comparison between library GNPS and query spectra of phytocomponents identified in *Turnera subulata* flower and leaf extracts by LC-MS/MS analyses

Comparison between library GNPS (bottom) and query spectra phytocomponents identified in **AFETS** (top). The structure of the phytocomponent identified is represented.

L-Tyrosine

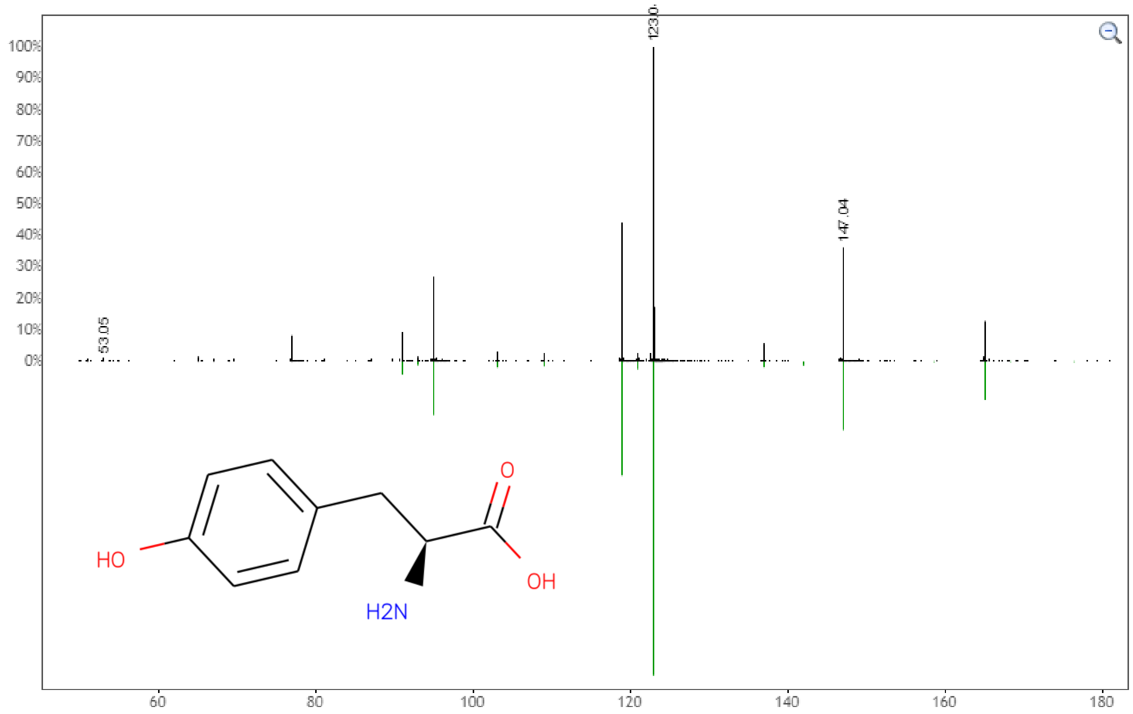

Phenylalanine

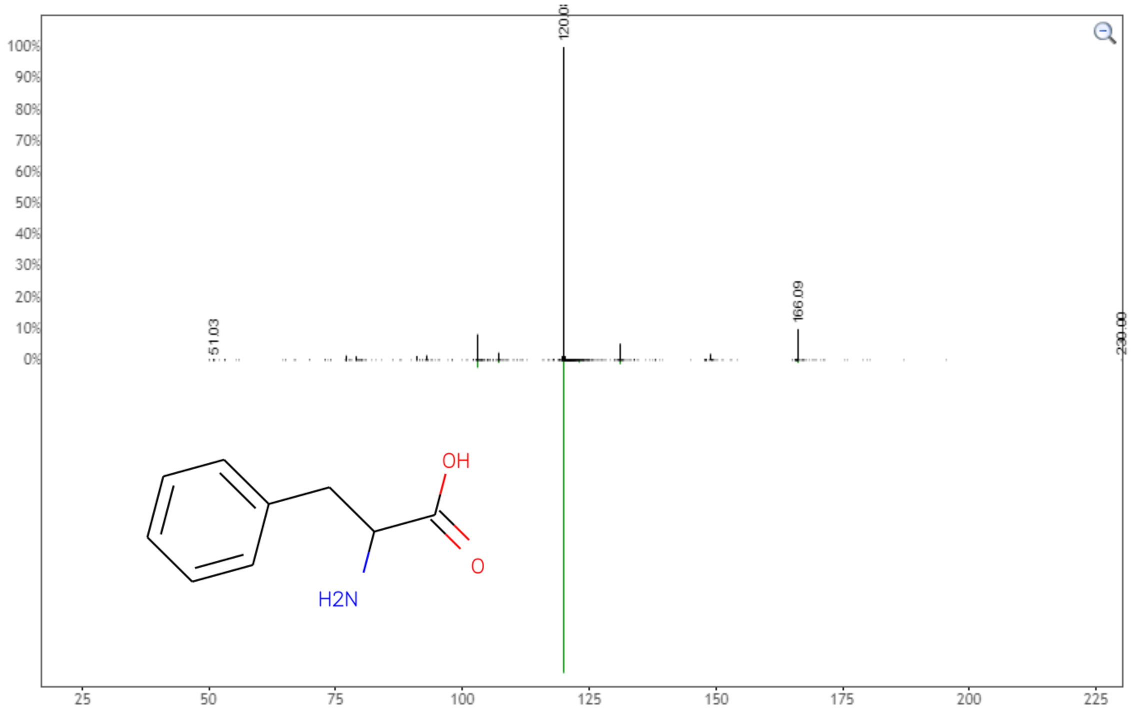

7-O-beta-glucopyranosyl-4’-hydroxy-5-methoxyisoflavone

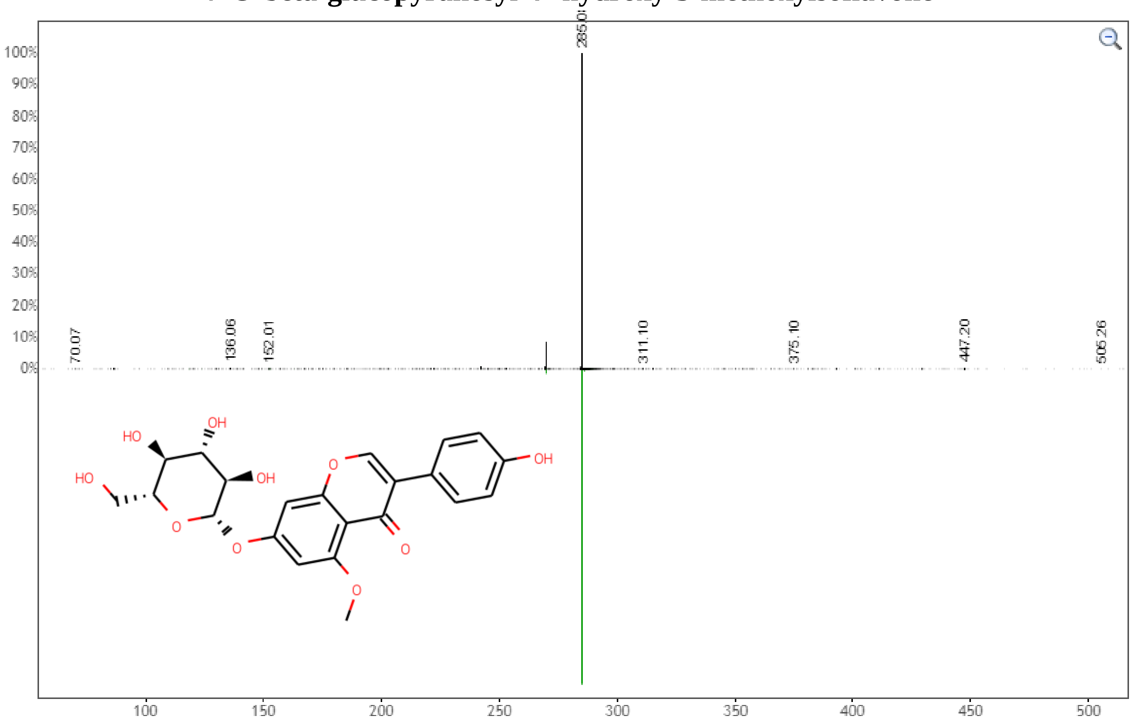

Vitexin-2-O-rhamnoside

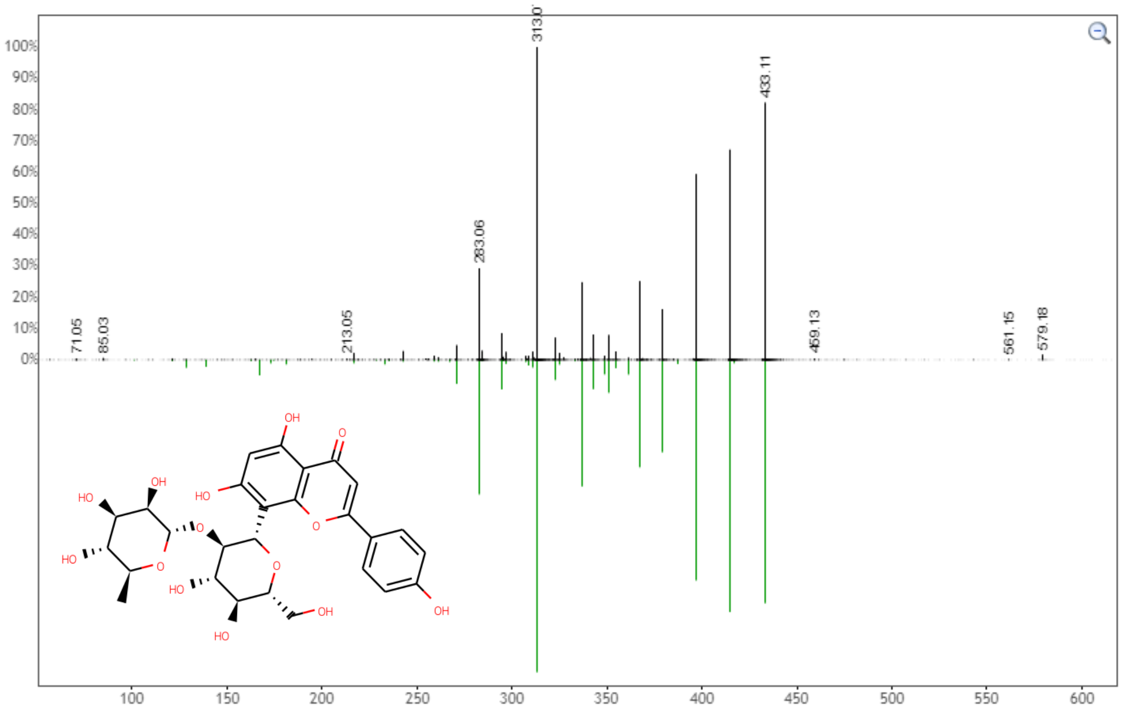

Comparison between library GNPS (bottom) and query spectra phytocomponents identified in **ALETS** (top). The structure of the phytocomponent identified is represented.

L-Tyrosine

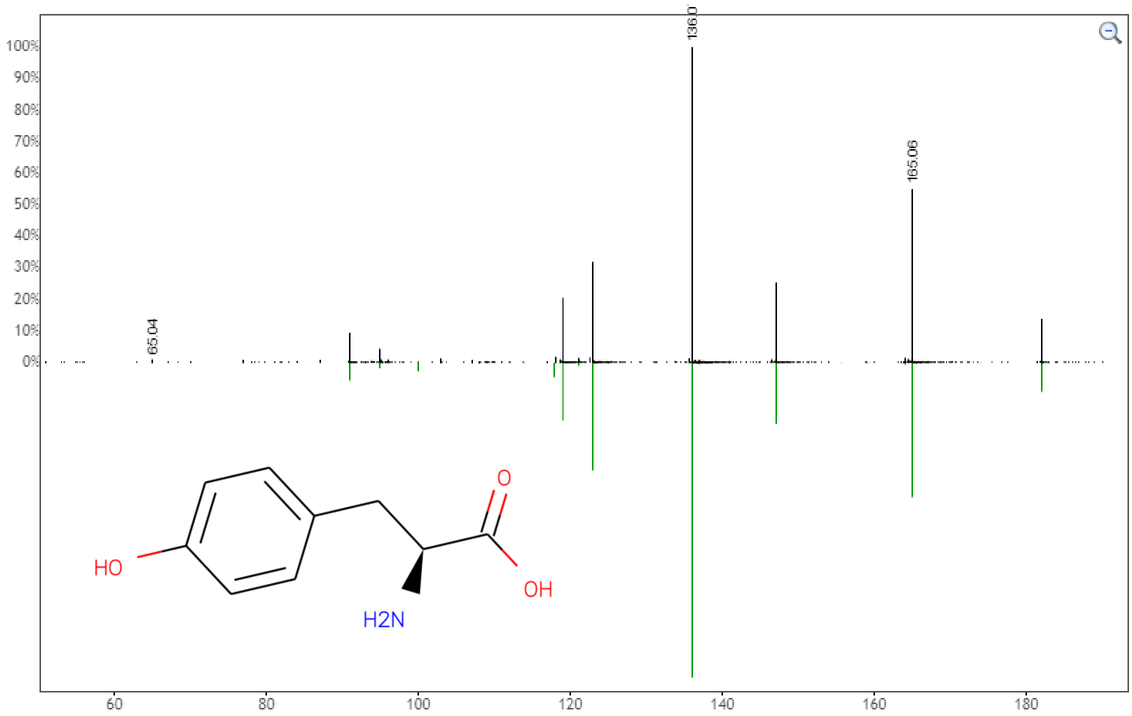

DL-Phenylalanine

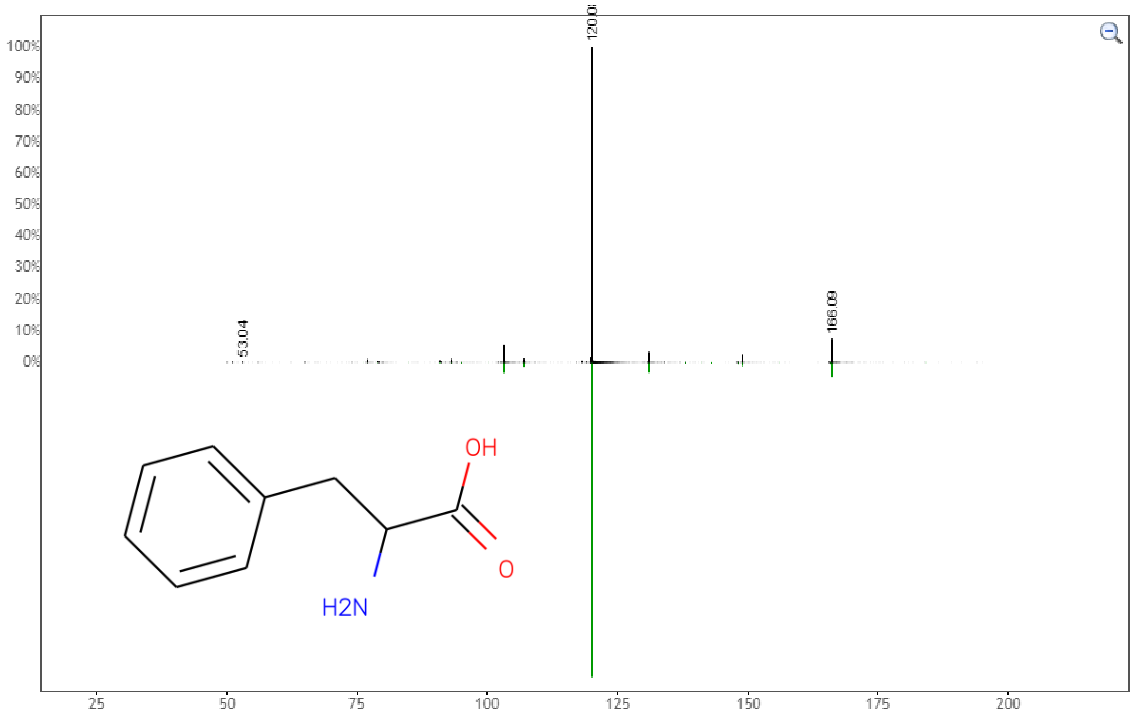

L-Tryptophan

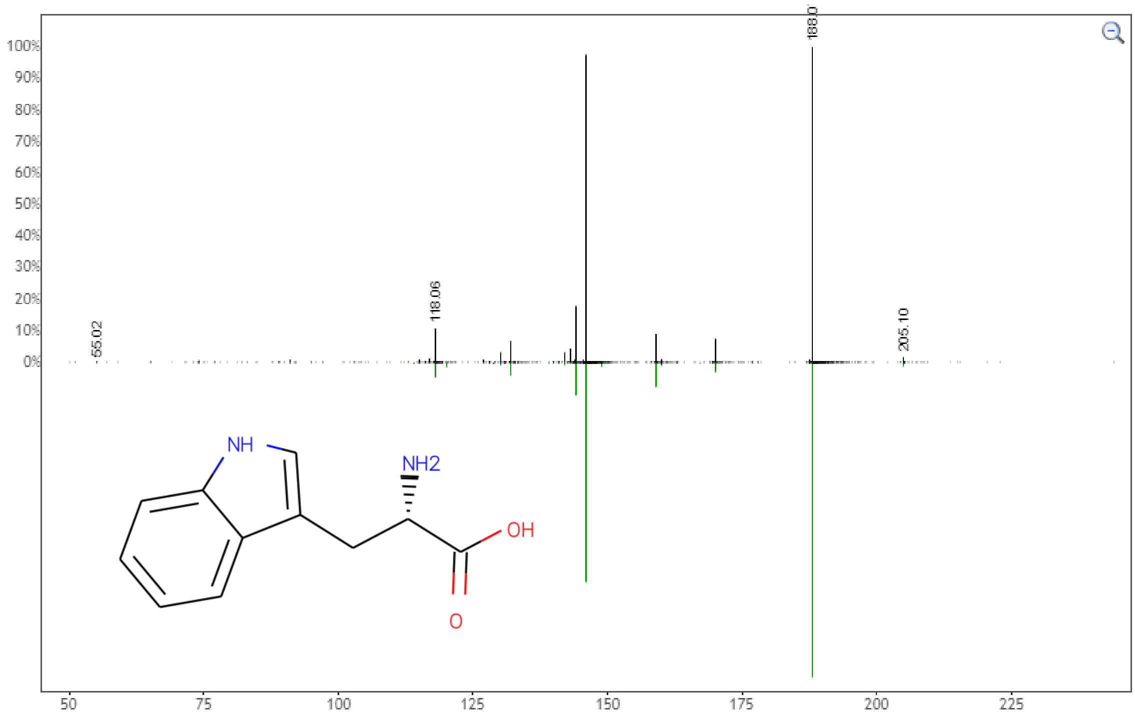

Ferulate/isoferulate

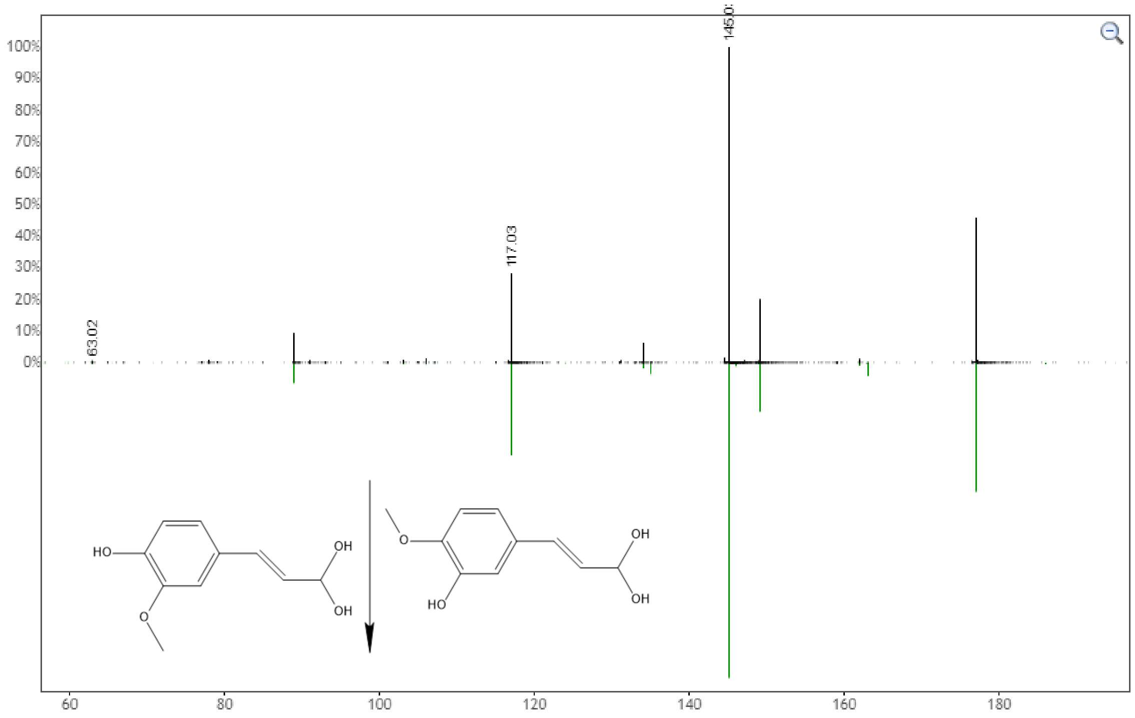

Vitexin-2-O-rhamnoside

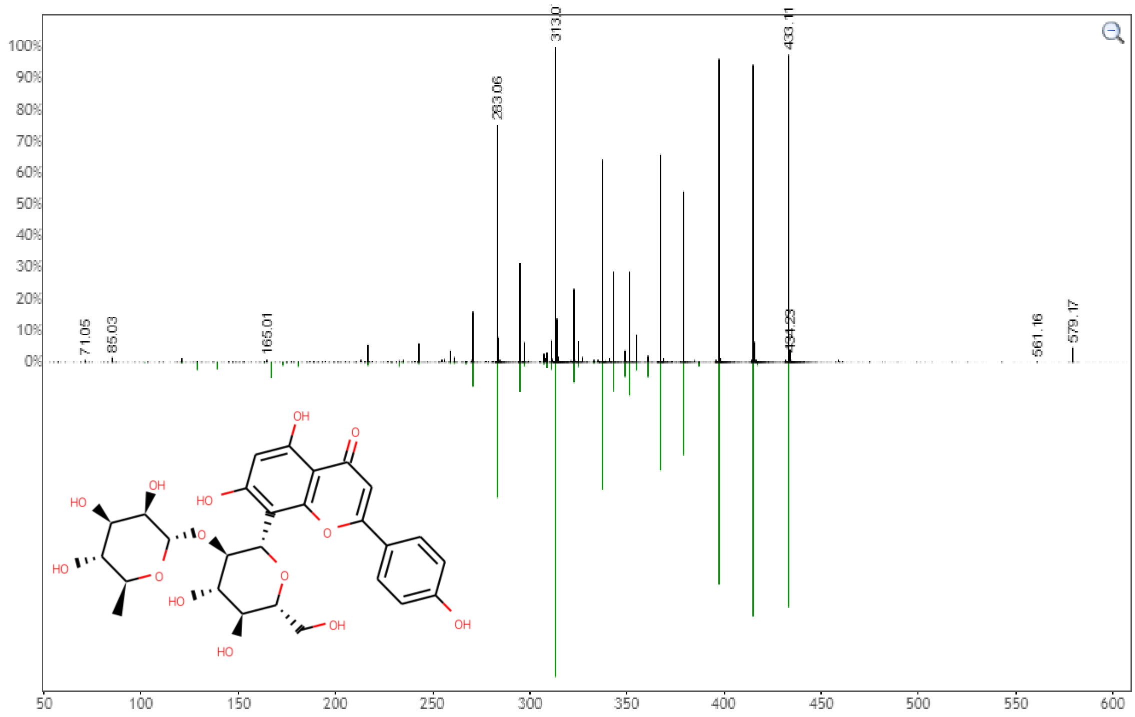

Comparison between library GNPS (bottom) and query spectra phytocomponents identified in **HEFTS** (top). The structure of the phytocomponent identified is represented.

DL-Octopamine

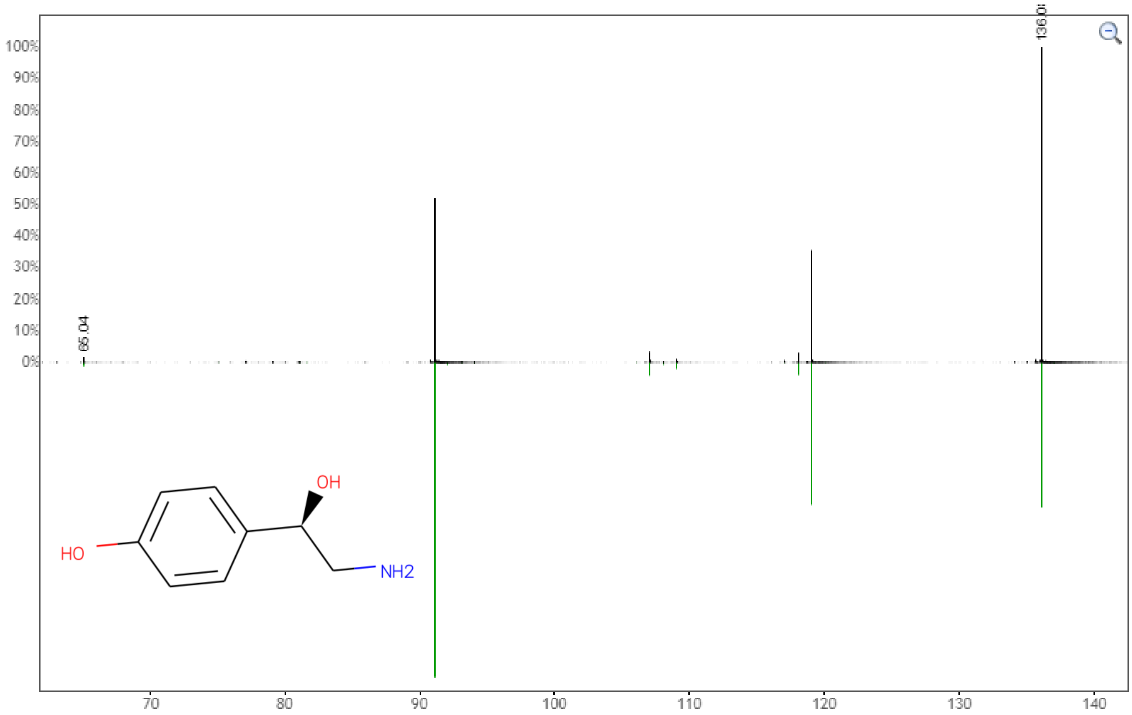

L-Tyrosine

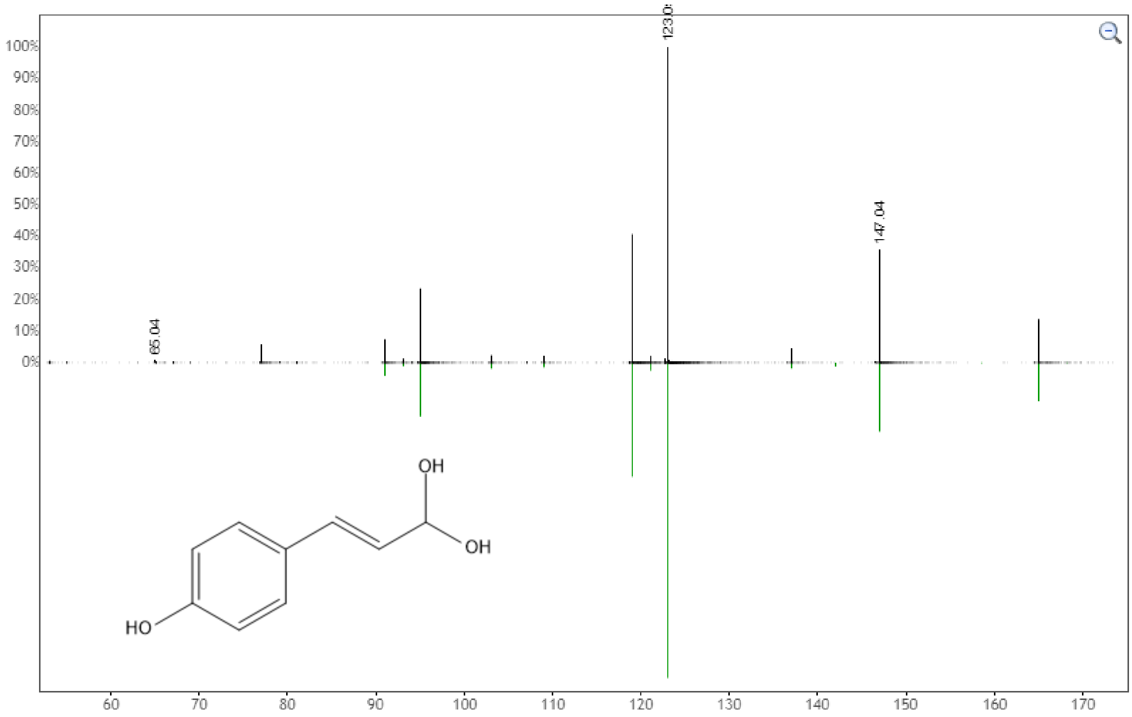

Phenylalanine

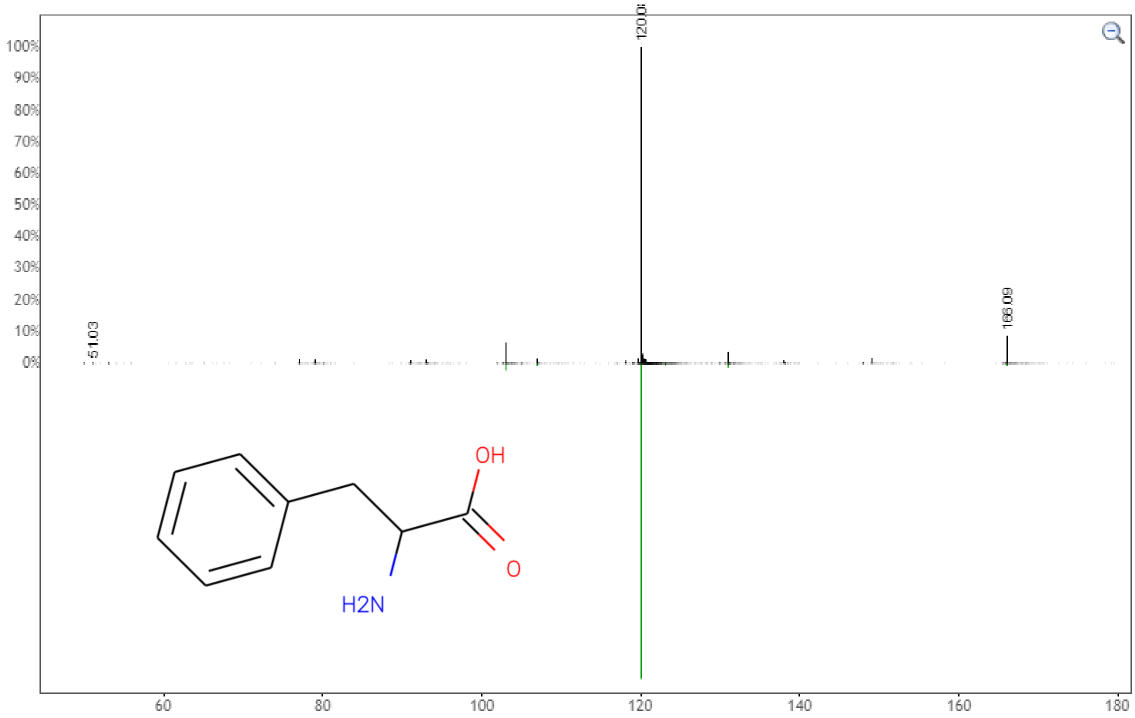

L-Tryptophan

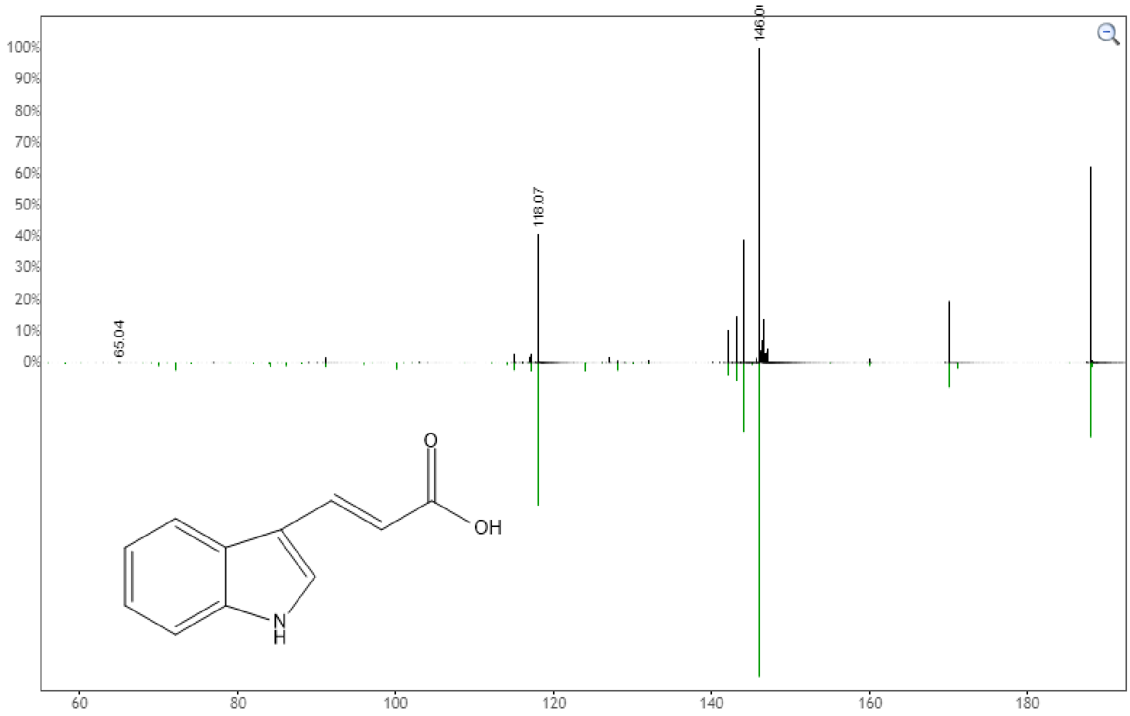

Adenosine, 5_-S-methyl-5_-thio-

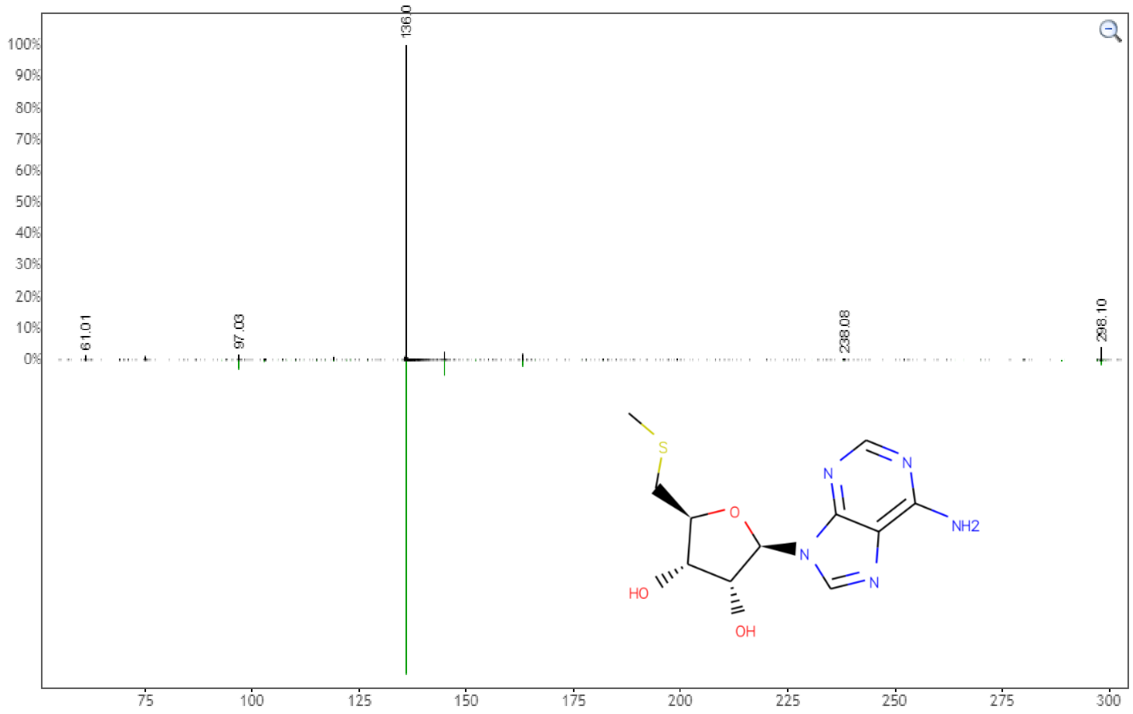

Ferulate

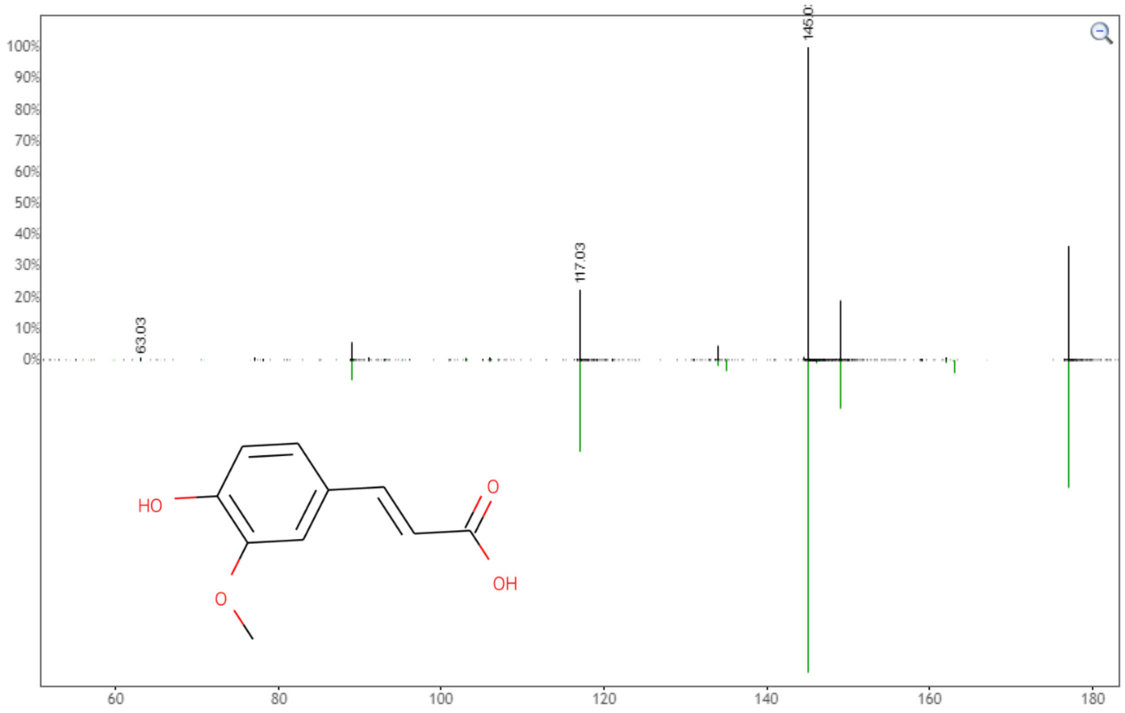

7-O-beta-glucopyranosyl-4’-hydroxy-5-methoxyisoflavone

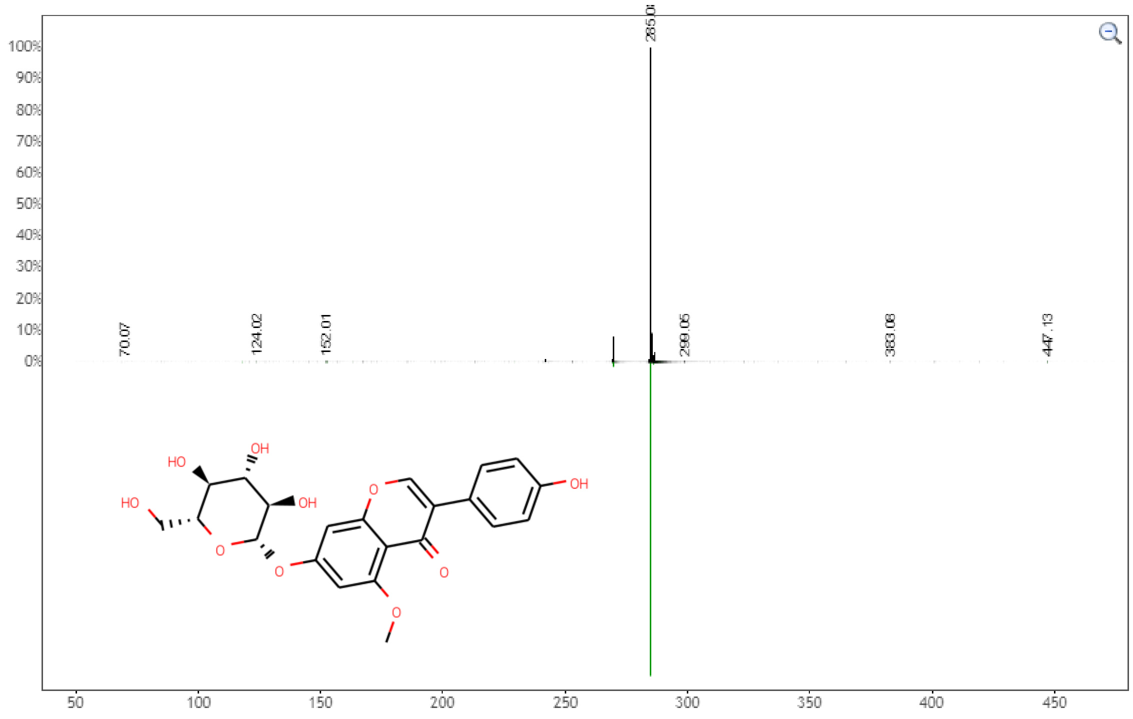

Vitexin-2-O-rhamnoside

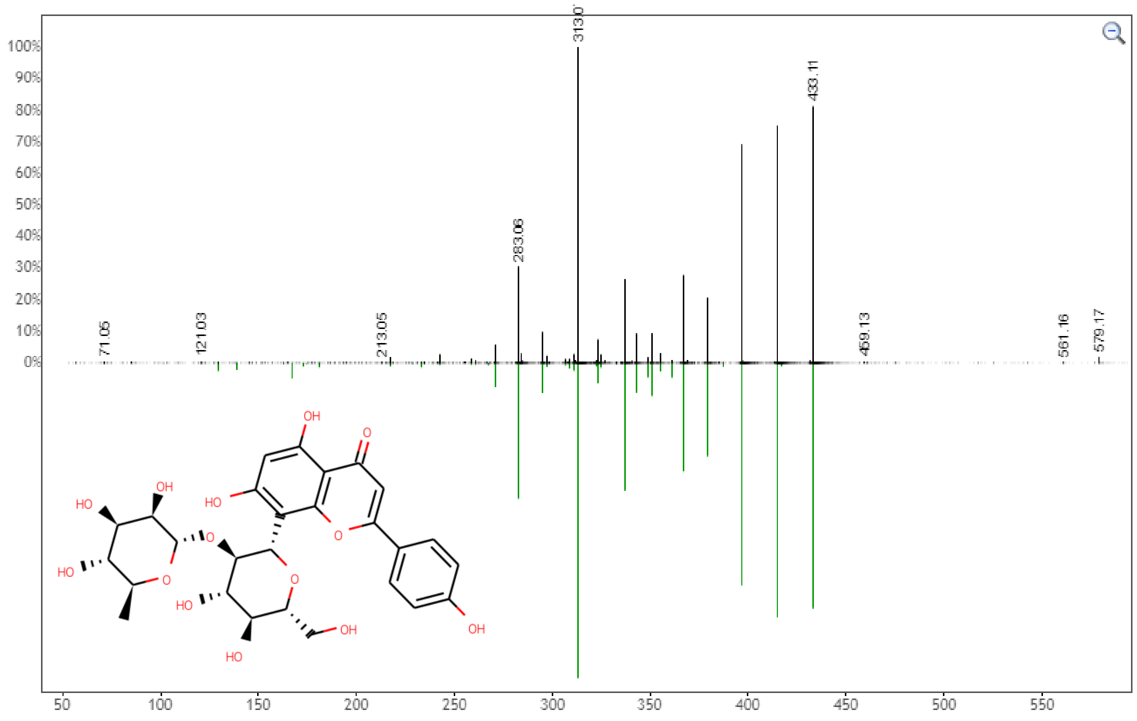

Comparison between library GNPS (bottom) and query spectra phytocomponents identified in **HELTS** (top). The structure of the phytocomponent identified is represented.

Phenylalanine

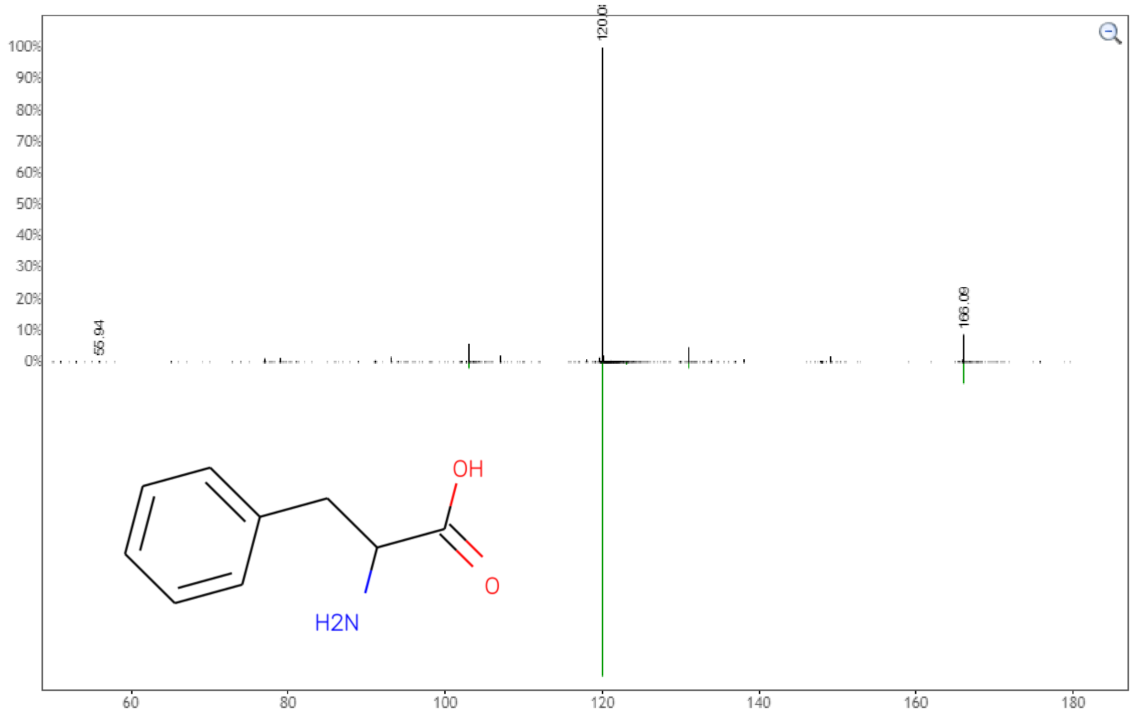

Ferulate

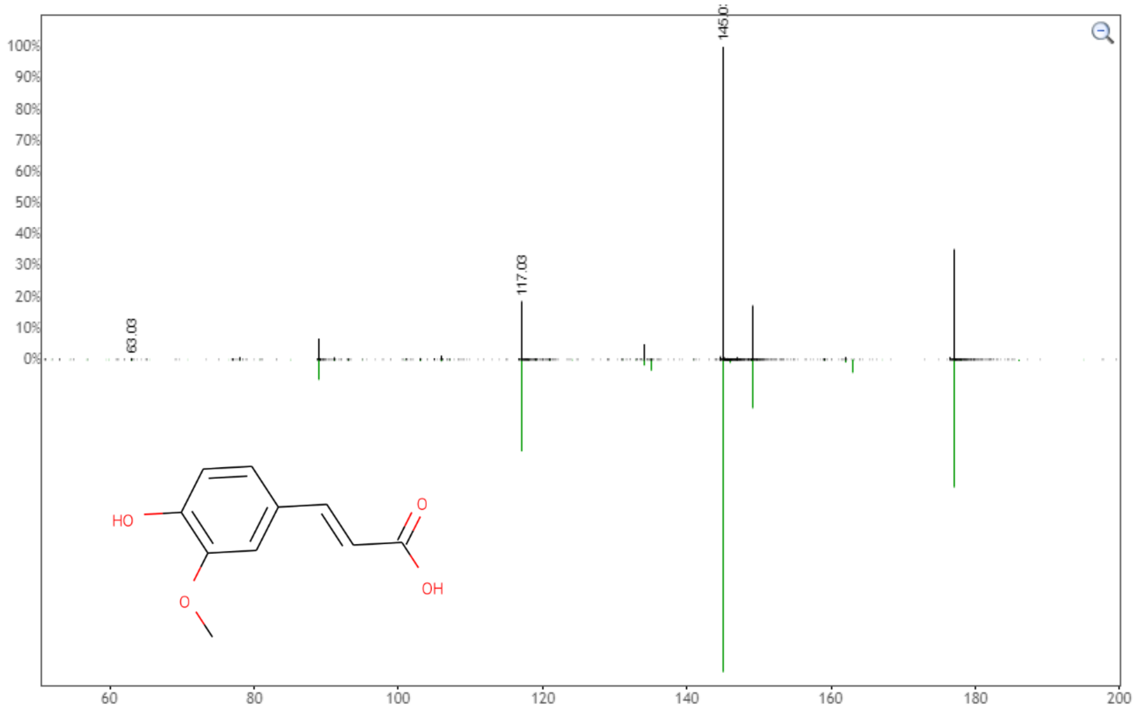

Vitexin-2-O-rhamnoside

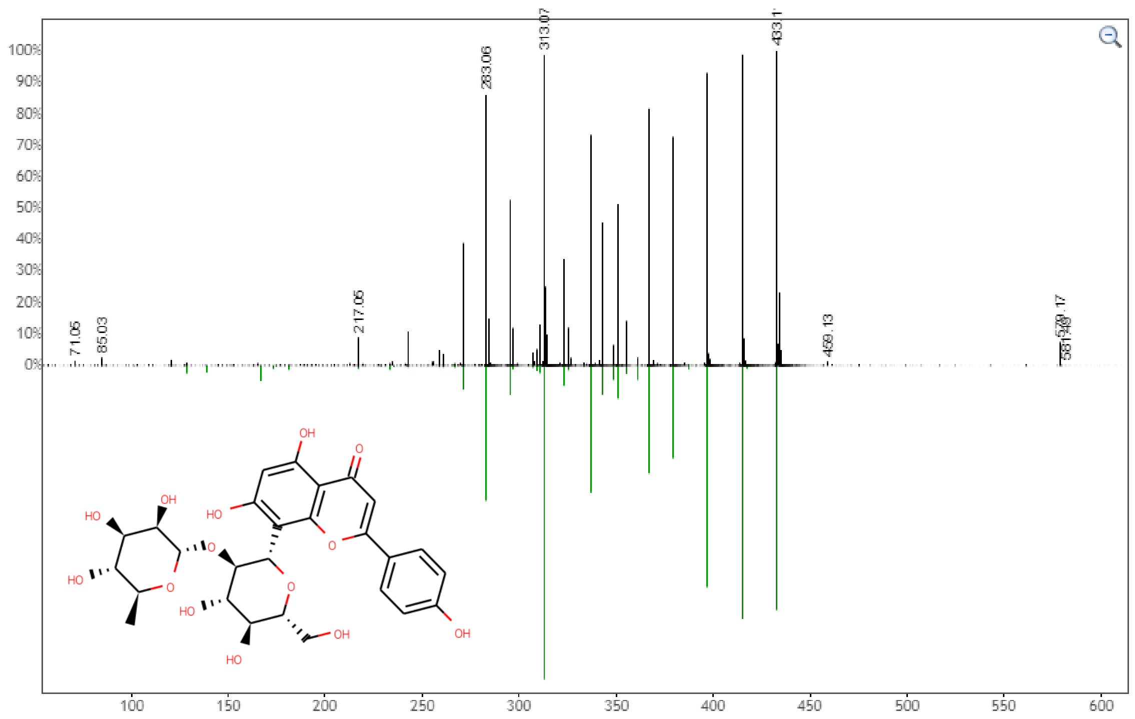

Loliolide

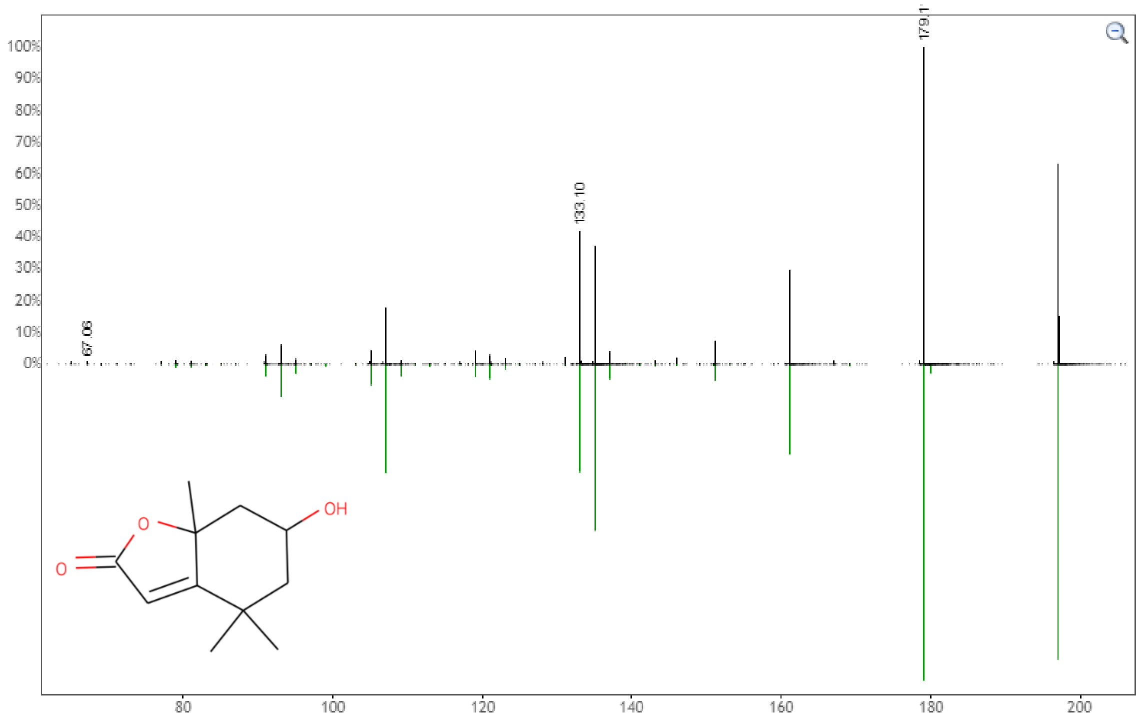

9S-Hydroxy-10E,12Z,15Z-octadecatrienoic acid

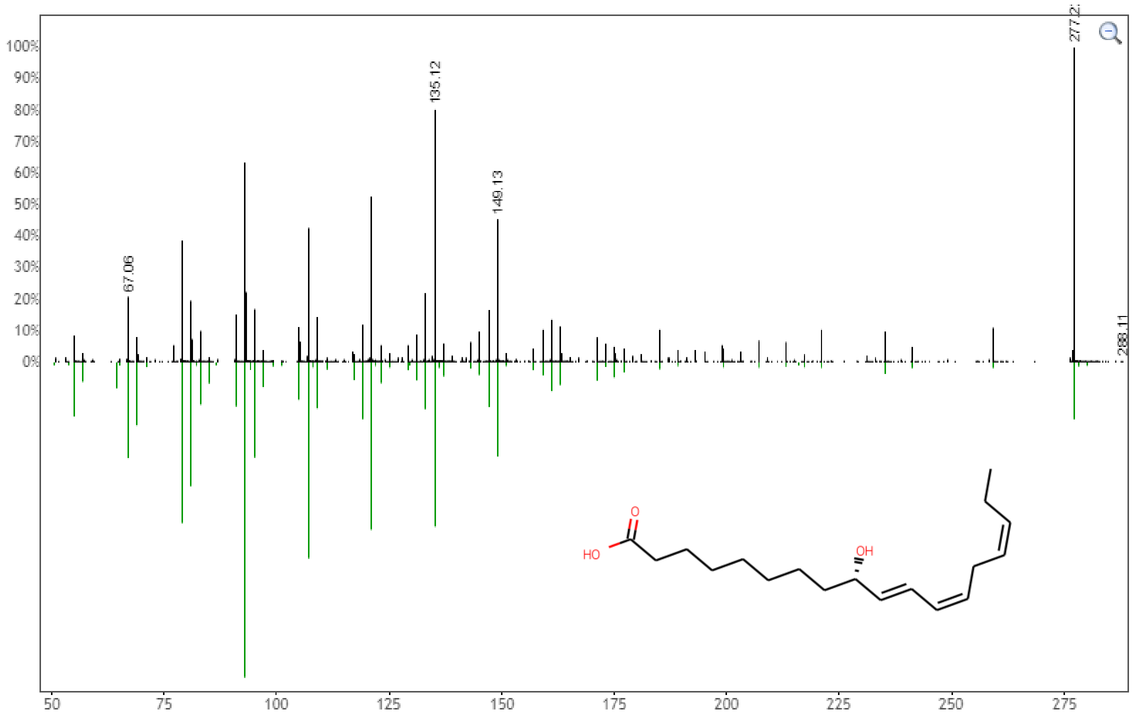

9(10)-EpOME

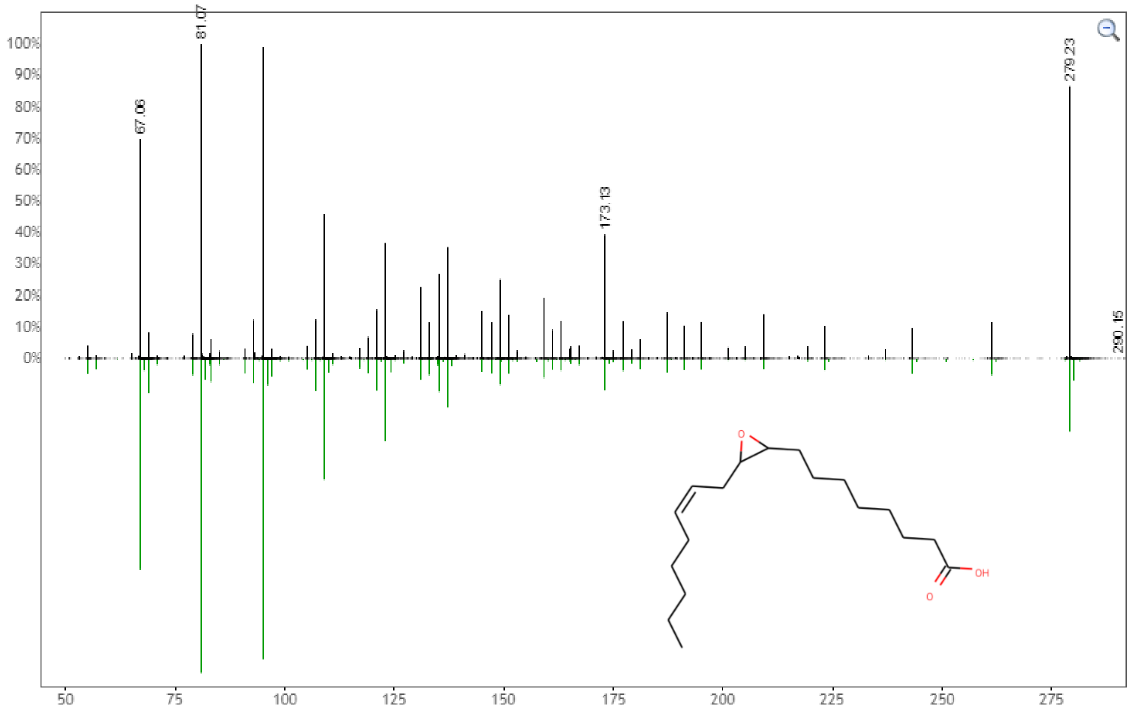

Stearidonic acid Ethyl ester

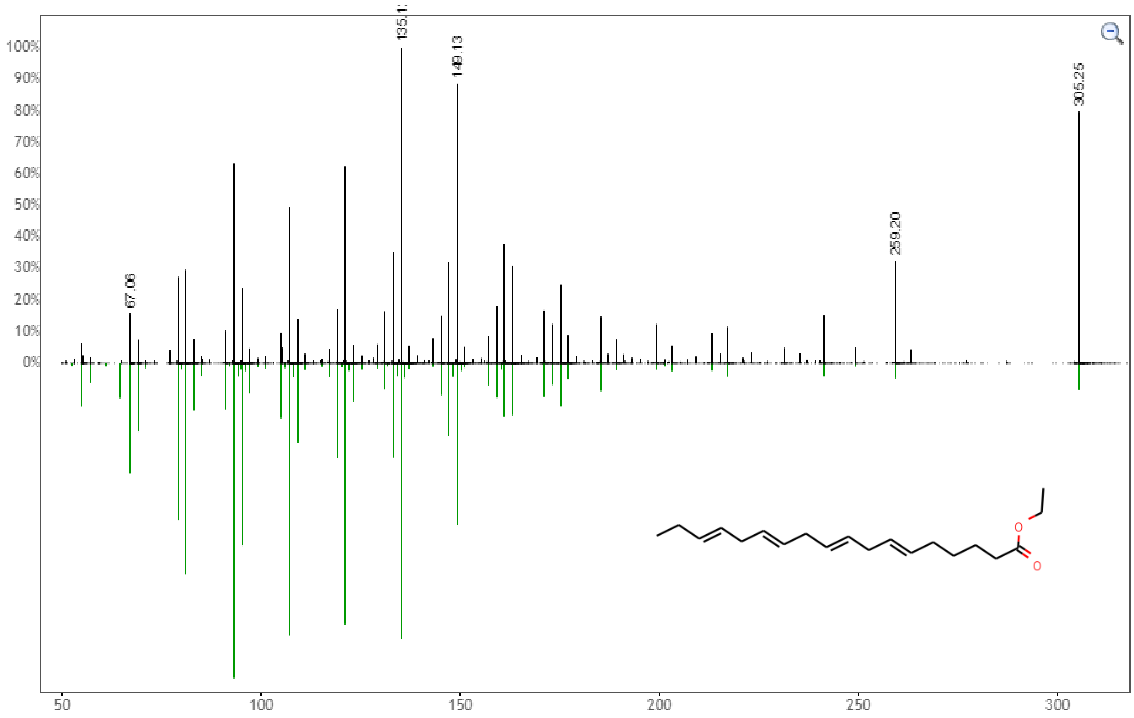

Pheophorbide A

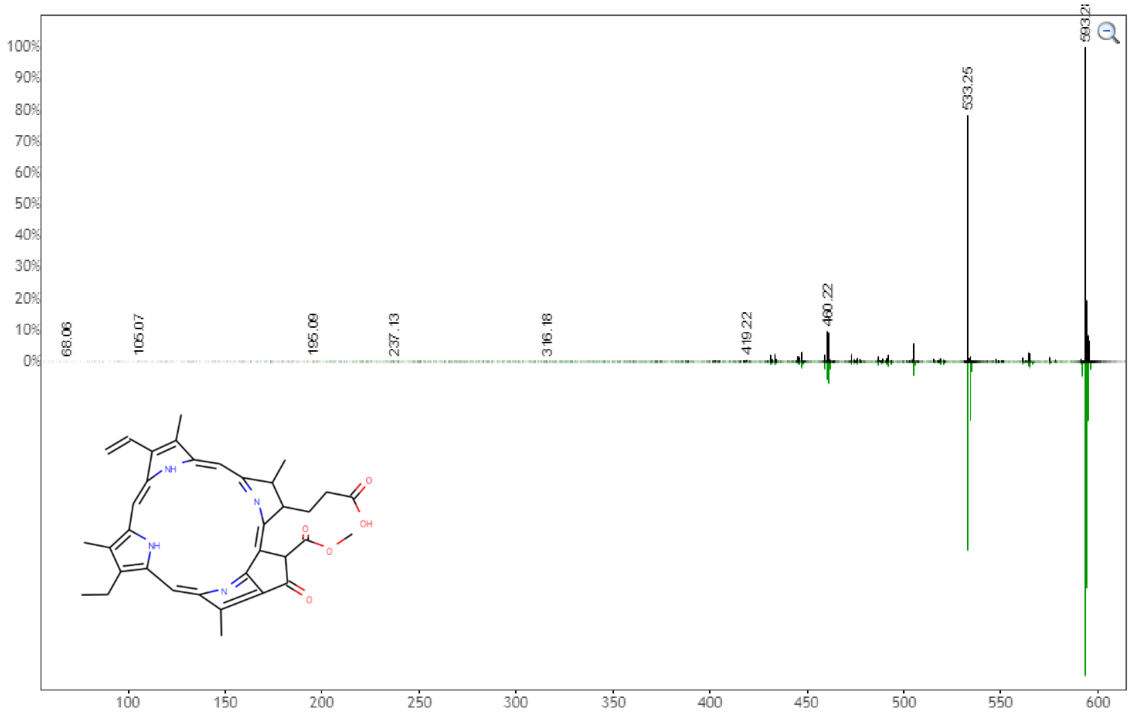

Supplement: Supplementary file 1 [file molecules-27-01084-s001.zip › molecules-1561005-supplementary.pdf]
